# Analysis of Synonymous Codon Usage Bias in Flaviviridae Virus

**DOI:** 10.1155/2019/5857285

**Published:** 2019-06-27

**Authors:** Huipeng Yao, Mengyu Chen, Zizhong Tang

**Affiliations:** College of Life Science, Sichuan Agriculture University, Ya'an 625014, Sichuan, China

## Abstract

**Background:**

Flaviviridae viruses are single-stranded, positive-sense RNA viruses, which threat human constantly mediated by mosquitoes, ticks, and sandflies. Considering the recent increase in the prevalence of the family virus and its risk potential, we investigated the codon usage pattern to understand its evolutionary processes and provide some useful data to develop the medications for most of Flaviviridae viruses.

**Results:**

The overall extent of codon usage bias in 65 Flaviviridae viruses is low with the average value of GC contents being 50.5% and the highest value being 55.9%; the lowest value is 40.2%. ENC values of Flaviviridae virus genes vary from 48.75 to 57.83 with a mean value of 55.56. U- and A-ended codons are preferred in the Flaviviridae virus. Correlation analysis shows that the positive correlation between ENC value and GC content at the third nucleotide positions was significant in this family virus. The result of analysis of ENC, neutrality plot analysis, and correlation analysis revealed that codon usage bias of all the viruses was affected mainly by natural selection. Meanwhile, according to correspondence analysis (CoA) based on RSCU and phylogenetic analysis, the Flaviviridae viruses mainly are made up of two groups, Group I (Yellow fever virus, Apoi virus, Tembusu virus, Dengue virus 1, and others) and Group II (West Nile virus lineage 2, Japanese encephalitis virus, Usutu virus, Kedougou virus, and others).

**Conclusions:**

All in, the bias of codon usage pattern is affected not only by compositional constraints but also by natural selection. Phylogenetic analysis also illustrates that codon usage bias of virus can serve as an effective means of evolutionary classification in Flaviviridae virus.

## 1. Introduction

All amino acids, except for methionine (Met) and tryptophan (Trp), are coded by more than one synonymous codon in the organism. The phenomenon that alternative synonymous codons do not occur equally is referred to as codon usage bias and this is a process of long-term accumulation. As an important evolutionary phenomenon, it is well known that synonymous codon usage bias exists in a wide range of species from prokaryotes to eukaryotes [1]. Compositional constraints and natural selection are thought to be two main factors influencing codon usage variation among the gene in different organisms [2, 3]. Flaviviridae viruses are single-stranded, positive-sense RNA viruses, which threat human constantly mediated by mosquitoes, ticks, and sandflies, such as Zika virus, Dengue virus, Yellow fever virus, Japanese encephalitis virus, and other viruses. Because their hosts are from the vertebrates and invertebrate, most of Flaviviridae viruses are related to some human diseases. For example, Dengue virus, Japanese encephalitis virus, and Zika virus are mediated by mosquitoes. Dengue virus contains four serotypes (DENV1 to DENV4) and its infection may cause symptoms from mild dengue fever to dengue hemorrhagic fever, even dengue shock syndrome [4] and stabilizing selection acts on the codon usage bias [5]. Spread of the Japanese encephalitis virus, reported from WHO, produced a total of 27, 059 patients during 2006~2009, out of which 86% were from China and India, 20~30% were caused to be fatal and 30~50% of the survivors were found to cause serious postinfection neurological sequelae and Japanese encephalitis virus has low codon usages bias influenced by both mutational pressure and natural selection [6]. Zika virus producing a number of microcephaly in Brazil is rapidly spreading to other parts of the world since 2015. Zika coding sequences have relatively conserved and genotype-specific evolution of codon usage bias [7]. Powassan virus, yellow fever virus, and spondweni virus are mediated by ticks. Powassan virus is a fatal, neurotropic virus, with a 671% rise in cases in the last 18 years, which has become an emerging danger worldwide [8]. Yellow fever virus can cause yellow fever which is endemic in many African and South American countries [9]. Spondweni virus can cause a self-limiting febrile illness characterized by headache, myalgia, nausea, and arthralgia similar to Zika virus infections [10]. Codon usage patterns of some members from the Flaviviridae viruses have been studied, such as Zika virus [7] and Dengue virus [5]. But the population codon usage characteristics of all Flaviviridae viruses have not been reported by now. Considering the recent increase in the prevalence of the family virus and its risk potential, we investigated the codon usage pattern to understand its evolutionary processes and provide some useful data to develop the medications for Flaviviridae viruses.

## 2. Materials and Methods

### 2.1. Genetic Material

The complete sequences of 65 Flaviviridae viruses were downloaded from NCBI (http://www.ncbi.nlm.nih.gov) and the detailed information about the viruses is listed in Table 1. The ORFs of the viruses were identified by DNAStar.

### 2.2. Nucleotide Composition Analysis

The following compositional properties were calculated for the coding sequences of the Flaviviridae virus genomes: (i) overall GC content; (ii) overall frequency of nucleotides (A%, C%, U%, and G%); (iii) frequency of each nucleotide at the third site of the synonymous codons (A_3S_%, C_3S_%, U_3S_%, and G_3S_%); (iv) frequency of nucleotides G + C at the third synonymous codon positions (GC_3S_%); (v) frequency of nucleotides G + C at the third codon positions (GC_3_) and the mean of the frequency of both G + C at the first and second position (GC_12_). The codons AUG and UGG are the only codons for Methionine and Tryptophan, respectively, and the termination codons UAA, UAG, and UGA do not encode any amino acids. Therefore, these five codons were excluded from the analysis. Nucleotide composition was calculated using the program CodonW 1.4.2 [11].

### 2.3. Effective Number of Codons (ENC) Analysis

ENC analysis was used to quantify the extent of the codon usage bias of viruses coding sequences, if regardless of the length of a given gene and the number of amino acids. The ENC values range from 20 to 61, in which the larger it is, the weaker the codon preference is. ENC of 20 indicates that there is only one of the synonymous codons for each amino acid and the value of the 61 means that all corresponding amino acids are coded by all synonymous codons equally. Generally, coding sequence has a codon bias significantly when the ENC value is less than or equal to 35 [7].

### 2.4. ENC-Plot Analysis

To determine the major factors affecting codon usage bias, an ENC-plot was analyzed with the ENC values plotted against the GC_3S_ values. If the points lie on or around the standard curve, the codon usage of given genes is only constrained by mutational pressure. Otherwise, the codon usage pattern is influenced by other factors, such as natural selection. The standard ENC values were calculated using the equation [12]:

(1) $$\begin{array}{c} {ENC}_{expected}=2+S+\frac{29}{\left(s^{2}+{\left(1-s\right)}^{2}\right)} \end{array}$$

“s” represents the given (G+C)_3S_% value

### 2.5. Neutrality Plot Analysis

The neutrality plot is also named neutral evolution analysis. It is used to compare the influences of mutation pressure and natural selection on the codon usage patterns of the virus coding sequences by plotting the GC_12_ values of the synonymous codons against the GC_3_ values [7]. The values of GC_12_ and GC_3_ of Flaviviridae virus were calculated by the EMBOSS CUSP program and then subjected to neutrality plot analysis.

### 2.6. Relative Synonymous Codon Usage (RSCU) Analysis

The RSCU values of the coding sequences were analyzed to gain the characters of synonymous codon usage pattern without the consideration of influence of the composition of amino acids and the size of coding region following a described method [7].The RSCU values were calculated as follows:

(2) $$\begin{array}{c} RSCU=\frac{x_{ij}}{\sum_{j}^{ni}x_{ij}}n_{i} \end{array}$$

x_*ij*_ represents the number of codons for the amino acid and* ni* represents the degenerate numbers of a specific synonymous codon that ranges from 1 to 61.

### 2.7. Correspondence Analysis

Correspondence analysis (CoA) is an effective method in identifying the major trends in the codon usage patterns among viruses coding sequences [5]. Each coding region was represented as 59-dimensional vector corresponding to RSCU value of each synonymous codon (excluding AUG, UGG, and stop codons). In this research, the CoA of Flaviviridae viruses were performed by CodonW.

### 2.8. Correlation Analysis

Correlation analysis was carried out to identify the factors influencing synonymous codon usage patterns by the statistical software SPSS22 [7]. The parameters of viruses were gained from the software EMBOSS CUSP program and CodonW.

### 2.9. Phylogenetic Analysis

The evolutionary processes of viruses significantly influence their codon usage pattern [13]. To determining the evolutionary relationship between different viruses, phylogenetic analysis based on the nucleotide sequences of coding region of viruses was performed using MEGA7 software.

## 3. Results 

### 3.1. Nucleotide Composition of 65 Flaviviridae Viruses

The nucleotide content of 65 Flaviviridae coding sequences was calculated. The results revealed that the A%, U%, G%, C%, and GC % were 27.03  ±  0.0236 (mean ± SD), 22.88  ±  0.0192, 28.49  ±  0.0253, 21.48  ±  0.0163, and 50.53  ±  0.0323, respectively. Further, for insight into its potential role on shaping the codon usage pattern, the base contents in the third position of Flaviviridae viruses were also calculated and A_3S_%, U_3S_%, G_3S_%, C_3S_%, and GC_3S_ % in these viruses were 33.11±0.0405 (mean ± SD), 34.54±0.0253, 27.01±0.0104, 29.14±0.0275, and 44.83±0.0508, respectively. It is clear that U_3S_% was distinctly high and G_3S_% was the lowest when compared to other base contents in the third position (Table 2). The result of CAI shows that in relation to E.human, the CAI values of Flaviviridae virus range from 0.673 to 0.740, with an average value of 0.714 and a SD of 0.0163 (Table 1).

### 3.2. The ENC-GC_*3*_s Plots Analysis

**Figure 1:**
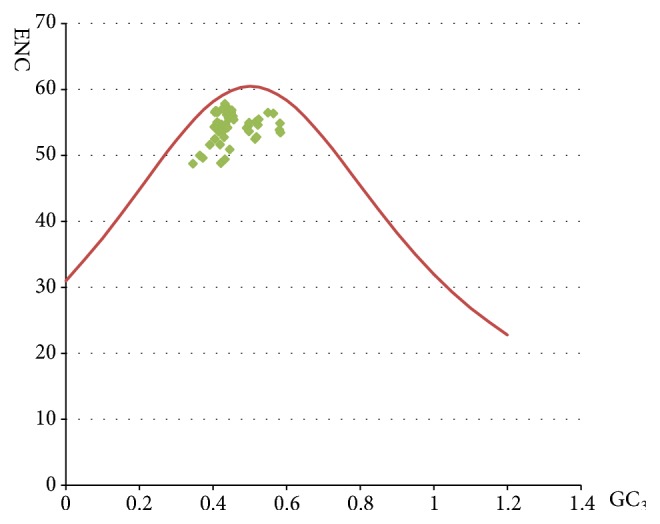
ENC-GC_3s_ plots.* ENC plotted against GC*_*3*S_. The red dotted line represents the expected curve derived from positions of strains when the codon usage was only determined by the GC_3S_ composition.

The mean value of the ENC values in the viruses was 54.58, the highest was 57.83, and the lowest was 48.75, in which the ENC values of 61 viruses were greater than 50, and that of 4 viruses was less than 50 (Table 2). It indicated that codon usage bias in Flaviviridae viruses is a little low. To investigate the factors affecting Flaviviridae virus codon usage bias, the ENC values were plotted against the GC_3S_ values. In ENC versus GC_3S_ graph, the curve represents the expected values of ENC with the only factor of mutation and the points represent the actual values of ENC of coding sequences in the Flaviviridae viruses (Figure 1). According to the ENC-GC_3S_ plots, all the viruses clustered together below the expected ENC curve, which indicated that in addition to mutation pressure, other factors, such as translational selection, also influence the codon usage pattern of Flaviviridae viruses coding sequences. [14].

### 3.3. The RSCU Analysis

**Figure 2:**
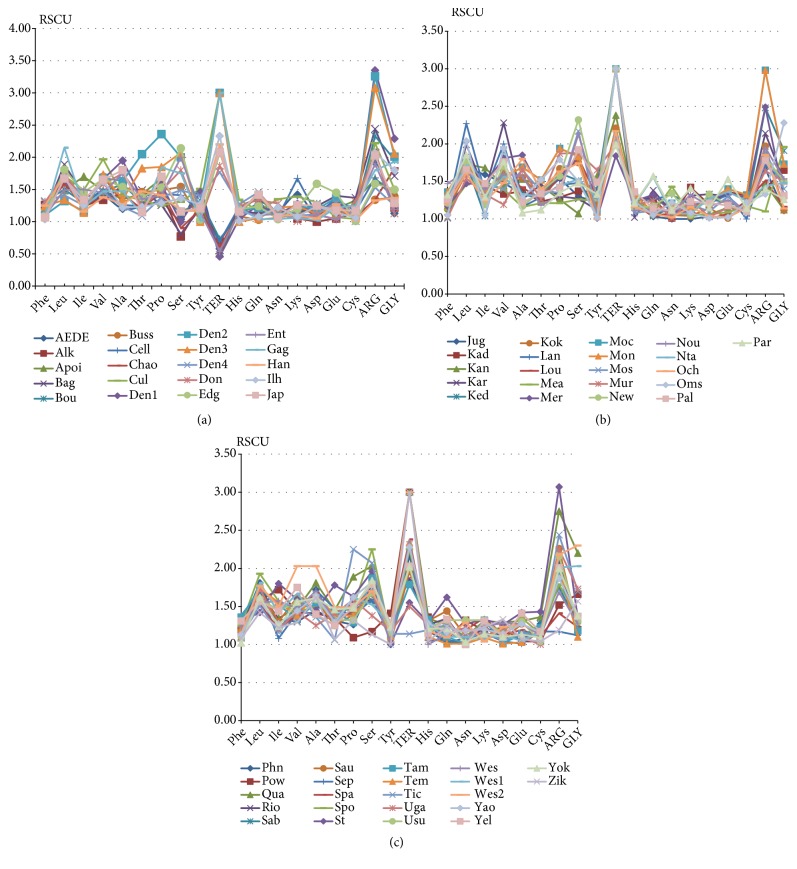
The optimal codons analysis. Analysis of relative synonymous codon usage in 65 Flaviviridae viruses. (a), (b), and (c) show the RSCU values of each optimal codon.

As shown in Table 3, most of the high-frequency codons are A/U-ended among the 18 amino acids in the viruses. For example, there are 53 viruses with high-frequency A/U-ended codons of Phenylalanine, accounting for 83.07%, those of Isoleucine accounting for 78.46%, and those of Valine accounting for 86.15%. In another word, Flaviviridae viruses prefer A/U-ended codons (Figure 2).

**Figure 3:**
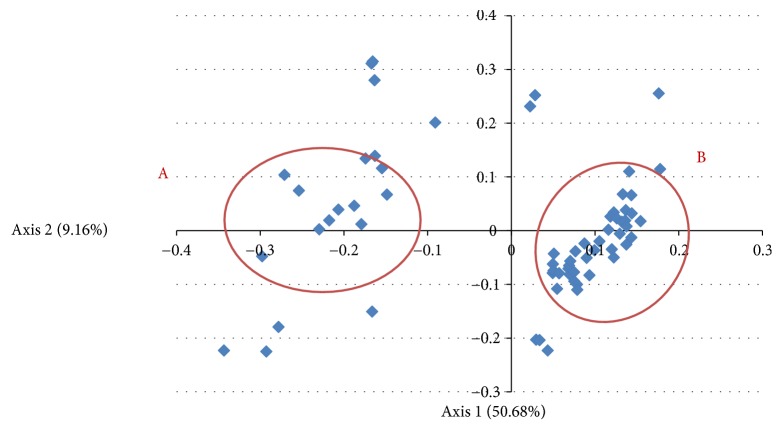
CoA on the RSCU values.* Correspondence analysis of the synonymous codon usage in Flaviviridae virus*. The analysis was based on the RSCU value of the 59 synonymous codons. The positions of each virus were described in the first two main-dimensional coordinates.

We performed CoA on the RSCU values, which revealed that the first, second, third, and fourth axis accounted for 50.68%, 9.16%, 3.51%, and 1.63% of the total variation, respectively. Thus, the codon usage bias could be mainly explained by the first axis and second axis values which were plotted to understand the distribution of synonymous codons usage patterns. Each point represents a virus and the closer the points are, the more similar the patterns of the viruses are. As shown in Figure 3, Flaviviridae viruses can be divided into two groups and the others, in which Group A includes Yellow fever virus, Apoi virus, Tembusu virus, Dengue virus 1, Wesselsbron virus and Group B includes West Nile virus lineage 2, Japanese encephalitis virus, Usutu virus, Kedougou virus.

### 3.4. Neutrality Plot Analysis

**Figure 4:**
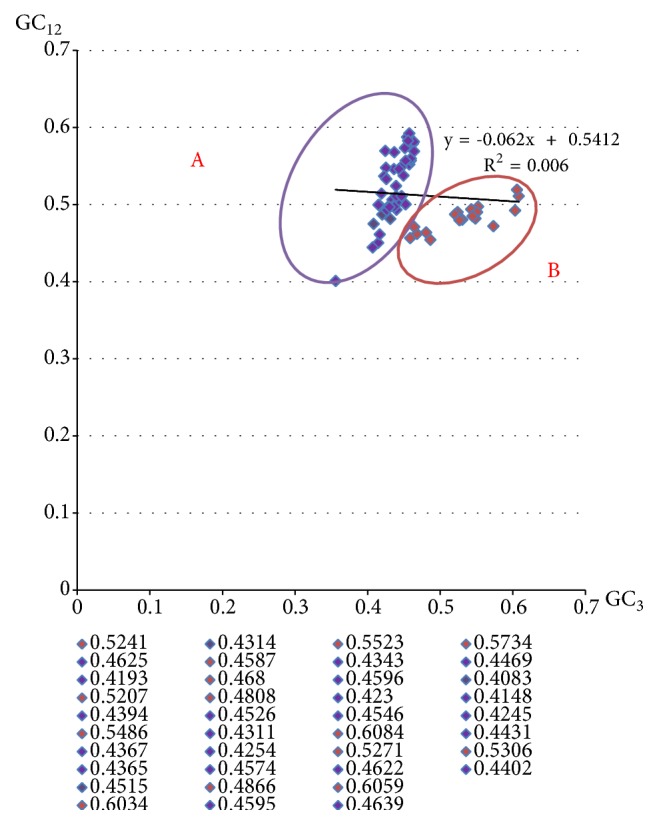
Neutrality plot analysis.* Neutrality plot analysis of thr 65 Flaviviridae viruses*. Neutrality plot analysis of the average GC content in the first and second position of the codons and the GC content in the third position.

In the neutrality plot analysis (Figure 4), a significant positive correlation was observed between the GC_12_ and GC_3_ values of Flaviviridae viruses (r^2^ = 0.06). The slope of the regression line was calculated to be 0.062 which indicated that the mutation pressure and natural selection were calculated to be 6.2% and 93.8%, respectively. It demonstrates the dominant influence of natural selection [15]. In addition, these viruses can be grouped into two clusters, Group A (Yellow fever virus, Apoi virus, Tembusu virus, Dengue virus 1, and others) and Group B (West Nile virus lineage 2, Japanese encephalitis virus, Usutu virus, Kedougou virus, and others) which is similar to the result of RSCU analysis.

### 3.5. Correlation Analysis

In Table 4, the ENC values had significant correlations with A%, C%, G%, A_3S_%, C_3S_%, and GC_3S_ %, respectively in Flaviviridae viruses. Additionally, GC_3S_ % had significant correlations with GC%. These data suggest that the nucleotide constraint influences synonymous codon usage.

ENC values have significant negative correlations with Gravy and Aroma. In addition, U_3S_ %, G_3S_%, C_3S_%, and GC_3S_% have significant negative correlations with Gravy values and A_3S_% have significant negative correlations with Aroma values. These results indicate that natural selection also influenced codon usage bias along with mutational pressure.

### 3.6. Phylogenetic Analysis of Flaviviridae Viruses

**Figure 5:**
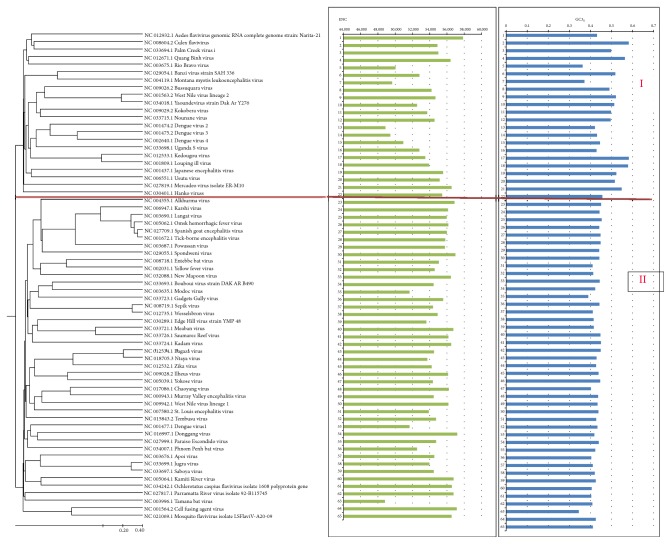
Phylogenetic analysis. Neighbor-joining analysis of Flaviviridae virus according to the phylogenetic analysis. Effective number of codons and GC_3S_ content for each species are also displayed.

To evaluate the effects of evolutionary processes on codon usage patterns, phylogenetic analysis was carried out. The results show that 65 Flaviviridae viruses can be divided into two groups (Figure 5), Group I and Group II. Group I includes Kedougou virus, Louping ill virus, West Nile virus lineage 2, and Yaounde virus, and the variation range of their GC3s content is not extensive (0.364 ≤ GC_3S_ ≤0.582). Group II includes Omsk hemorrhagic fever, Alkhurma virus, Tick-borne encephalitis virus, Spanish goat encephalitis virus. And, the variation range of their GC_3S_ content is relatively smaller (0.345 ≤ GC_3S_ ≤ 0.454, respectively). These results suggest that the closer the evolution of species classification, the more similar their codon usage bias

## 4. Discussion

Study of codon usage patterns of viruses can reveal more useful information about overall viral survival, fitness, and evolution [6]. In this research, the majority of Flaviviridae viruses have a weak codon bias with the mean ENC value of 54.58. And this is in accordance with some earlier studies on codon usage bias of Tembusu virus and West Nile virus which has a low codon usage bias [16–18]. According to the calculation results of CodonW (Table 2), the content of A and G is the highest and RSCU analysis indicates that Flaviviridae viruses prefer A/U-ended codons.

Linking to other RNA viruses, such as polioviruses, H5N1 influenza virus, and SARS-covs with the mean ENC values of 53.75, 50.91, and 48.99 [19–21], respectively, we conjecture that the weak codon bias in RNA virus is advantageous to replicate efficiently in host cells [22]. As ENC-GC_3S_ plots analysis shows, mutational pressure and other factors shaped the codon usage patterns of Flaviviridae viruses, which is similar to hepatitis C virus [22]. In fact, Hongju et al. have previously reported that the codon usage bias of ZIKV is weak and the influencing factors of the patterns are not only mutation pressure, but also translational selection, aromaticity, and hydrophobicity [14]. Although in previous studies [14, 23] on Zika virus, it is observed there were greater frequencies of A_3S_/G_3S_ than U_3S_. There were some viruses showing contrary characteristics; for example, Aedes flavivirus U_3S_% was 0.2994 and G_3S_% was 0.279; Alkhurma virus U_3S_ was % 0.3617 and G_3S_% was 0.2773. By comprehensive analysis of all results, it can be found that overall U_3S_% was more and G_3S_% was lowest. Since Flaviviridae viruses prefer A/U-ended codons and A_3S_% has a remarkable correlation with ENC (Table 3), we think that compositional constraint shaping the synonymous codon bias was from the content of nucleotides A and U on the third codon position. This result was different from many reports in which compositional constraints influencing codon usage bias are from G and C contents (Zhou* et al. *2004) [20, 24]. In addition, it can be found that the correlations of both Gravy values and Aroma values with ENC values are significant, which indicates the role of natural selection in shaping the codon usage patterns of the Flaviviridae viruses [6]. Besides, the codon usage patterns of this family were influenced by nature selection which dominates 93.8% and mutation pressure which dominates 6.2% (Figure 4).

In CoA-RSCU analysis, the Flaviviridae viruses can be divided into two groups and the others. The viruses which have similar codon usage patterns are clustered together. It is similar to the result from Neutrality plot analysis and the phylogenetic tree. All in, it is found that Yellow fever virus, Apoi virus, Tembusu virus, and Dengue virus 1 always clustered together.

In summary, combining the nucleotide composition analysis, ENC-plot analysis, and correlation analysis, it is clear that both mutation pressure and nature selection influence the codon usage patterns of Flaviviridae viruses. In addition, most of the Flaviviridae viruses can also be classified into two categories according to the findings of the CoA-RSCU, neutrality plot analysis, and phylogenetic analysis. Codon usage patterns were similar between different virus species in same group.

## 5. Conclusion

In this study, the majority of Flaviviridae viruses have a weak codon usage bias which help to adapt to the diverse host or the varied environment. The Flaviviridae viruses can also be classified into two groups according their codon usage patterns. Their codon usage patterns were influenced by nature selection which dominates 93.8% and mutation pressure which dominates 6.2%. The information from this research may not only help to understand the evolution of Flaviviridae virus, but also have potential value for developing the virus vaccines.

## Figures and Tables

**Table 1:** The basic information of Flaviviridae.

Organism/Name	Accession id	ENC	GC_3S_	GC_12_	GC_3_	Gravy	Aromo	CAI
Aedes flavivirus	NC_012932.1	58.97	0.502	0.501	0.5241	-0.057	0.080	0.682
Alkhurma hemorrhagic fever virus	NC_004355.1	56.85	0.451	0.553	0.4625	-0.797	0.062	0.715
Apoi virus	NC_003676.1	54.49	0.411	0.497	0.4193	-0.751	0.075	0.713
Bagaza virus	NC_012534.1	54.46	0.429	0.508	0.5207	-1.006	0.065	0.73
Banzi virus	NC_029054.1	52.79	0.518	0.504	0.4394	-0.112	0.082	0.74
Bouboui virus	NC_033693.1	54.42	0.423	0.495	0.5486	-0.882	0.073	0.722
Bussuquara virus	NC_009026.2	54.19	0.491	0.499	0.4367	-0.151	0.082	0.712
Cell fusing agent virus	NC_001564.2	57.10	0.426	0.522	0.4365	-0.599	0.078	0.683
Chaoyang virus	NC_017086.1	56.19	0.437	0.495	0.4515	-0.894	0.076	0.724
Culex flavivirus	NC_008604.2	54.87	0.582	0.529	0.6034	-0.019	0.095	0.702
Dengue virus 1	NC_001477.1	52.15	0.424	0.476	0.4314	-1.016	0.065	0.742
Dengue virus 2	NC_001474.2	48.85	0.421	0.457	0.4587	-0.226	0.077	0.701
Dengue virus 3	NC_001475.2	49.43	0.432	0.464	0.468	-0.219	0.078	0.696
Dengue virus 4	NC_002640.1	50.90	0.445	0.470	0.4808	-0.188	0.081	0.703
Donggang virus	NC_016997.1	57.18	0.440	0.501	0.4526	-0.756	0.097	0.714
Edge Hill virus	NC_030289.1	53.61	0.416	0.491	0.4311	-0.806	0.088	0.728
Entebbe bat virus	NC_008718.1	55.04	0.411	0.516	0.4254	-0.905	0.060	0.71
Gadgets Gully virus	NC_033723.1	55.54	0.443	0.532	0.4574	-0.872	0.068	0.735
Hanko virus	NC_030401.1	55.43	0.456	0.465	0.4866	-0.021	0.100	0.675
Ilheus virus	NC_009028.2	56.12	0.447	0.535	0.4595	-0.968	0.060	0.715
Japanese encephalitis virus	NC_001437.1	55.49	0.524	0.516	0.5523	-0.209	0.081	0.72
Jugra virus	NC_033699.1	53.93	0.421	0.493	0.4343	-0.873	0.069	0.718
Kadam virus	NC_033724.1	56.45	0.449	0.539	0.4596	-0.851	0.066	0.729
Kamiti River virus	NC_005064.1	56.76	0.407	0.511	0.423	-0.666	0.081	0.681
Karshi virus	NC_006947.1	56.10	0.444	0.555	0.4546	-0.804	0.064	0.717
Kedougou virus	NC_012533.1	53.46	0.583	0.544	0.6084	-0.125	0.081	0.74
Kokobera virus	NC_009029.2	53.67	0.498	0.496	0.5271	-0.161	0.083	0.711
Langat virus	NC_003690.1	55.94	0.454	0.555	0.4622	-0.803	0.054	0.723
Louping ill virus	NC_001809.1	53.88	0.580	0.548	0.6059	-0.150	0.080	0.736
Meaban virus	NC_033721.1	56.71	0.448	0.556	0.4639	-0.894	0.069	0.721
Mercadeo virus	NC_027819.1	56.50	0.549	0.506	0.5734	-0.059	0.101	0.699
Modoc virus	NC_003635.1	51.61	0.391	0.467	0.4469	-0.701	0.092	0.727
Montana myotis leukoencephalitis virus	NC_004119.1	49.63	0.372	0.439	0.4083	-0.127	0.090	0.694
Mosquito flavivirus	NC_021069.1	56.52	0.412	0.534	0.4148	-0.667	0.066	0.673
Murray Valley encephalitis virus	NC_000943.1	54.42	0.434	0.498	0.4245	-0.925	0.086	0.734
New Mapoon virus	NC_032088.1	56.42	0.445	0.530	0.4431	-0.983	0.067	0.714
Nounane virus	NC_033715.1	54.51	0.496	0.497	0.5306	-0.138	0.088	0.71
Ntaya virus	NC_018705.3	53.72	0.427	0.493	0.4402	-0.904	0.070	0.728
Ochlerotatus caspius flavivirus	NC_034242.1	56.55	0.405	0.483	0.4159	-0.612	0.092	0.696
Omsk hemorrhagic fever virus	NC_005062.1	56.13	0.443	0.545	0.4534	-0.888	0.061	0.72
Palm Creek virus	NC_033694.1	54.98	0.498	0.495	0.5267	0.008	0.095	0.682
Paraiso Escondido virus	NC_027999.1	54.68	0.423	0.487	0.4398	-0.804	0.082	0.723
Parramatta River virus	NC_027817.1	56.73	0.409	0.485	0.4202	-0.646	0.093	0.693
Phnom Penh bat virus	NC_034007.1	52.49	0.405	0.461	0.4168	-0.638	0.106	0.72
Powassan virus	NC_003687.1	55.72	0.442	0.545	0.4515	-0.799	0.061	0.718
Quang Binh virus	NC_012671.1	56.38	0.564	0.523	0.4369	-0.008	0.094	0.698
Rio Bravo virus	NC_003675.1	50.00	0.364	0.432	0.4070	-0.117	0.095	0.689
Saboya virus	NC_033697.1	54.45	0.426	0.492	0.4370	-0.832	0.077	0.724
Saumarez Reef virus	NC_033726.1	56.15	0.450	0.548	0.4641	-0.919	0.071	0.728
Sepik virus	NC_008719.1	54.34	0.412	0.483	0.4262	-0.811	0.083	0.717
Spanish goat encephalitis virus	NC_027709.1	55.92	0.449	0.559	0.4575	-0.862	0.060	0.724
Spondweni virus	NC_029055.1	56.95	0.444	0.532	0.4526	-0.938	0.058	0.715
St. Louis encephalitis virus	NC_007580.2	53.85	0.428	0.507	0.4395	-0.988	0.067	0.727
Tamana bat virus	NC_003996.1	48.75	0.345	0.402	0.3560	-0.674	0.126	0.704
Tembusu virus	NC_015843.2	54.69	0.434	0.503	0.4469	-0.894	0.078	0.727
Tick-borne encephalitis virus	NC_001672.1	55.79	0.448	0.552	0.4560	-0.777	0.064	0.722
Uganda S virus	NC_033698.1	52.78	0.430	0.469	0.4643	-0.111	0.085	0.701
Usutu virus	NC_006551.1	55.13	0.516	0.511	0.5428	-0.165	0.083	0.721
Wesselsbron virus	NC_012735.1	54.89	0.415	0.487	0.4303	-0.801	0.086	0.717
West Nile virus 1	NC_009942.1	56.15	0.438	0.522	0.5509	-0.973	0.061	0.714
West Nile virus 2	NC_001563.2	54.62	0.522	0.510	0.4432	-0.150	0.083	0.729
Yaounde virus	NC_034018.1	52.50	0.514	0.505	0.5447	-0.160	0.083	0.721
Yellow fever virus	NC_002031.1	54.54	0.413	0.509	0.4257	-0.822	0.084	0.731
Yokose virus	NC_005039.1	54.33	0.403	0.483	0.4151	-0.875	0.073	0.718
Zika virus	NC_012532.1	54.21	0.439	0.520	0.4495	-0.904	0.063	0.728

**Table 2:** Nucleotide contents in ORFs of 66 Flaviviridae virus genomes.

virus strain	A	U	G	C	A_3S_	T_3S_	G_3S_	C_3S_	GC
Aedes flavivirus	0.2528	0.2467	0.2621	0.2384	0.2991	0.2994	0.279	0.3412	0.501
Alkhurma virus	0.2436	0.2157	0.371	0.2237	0.2976	0.3617	0.2773	0.2729	0.553
Apoi virus	0.2763	0.2754	0.2754	0.2075	0.3367	0.3906	0.2441	0.2722	0.497
Bagaza virus	0.2916	0.2125	0.2929	0.2129	0.3307	0.3727	0.2501	0.2893	0.508
Banzi virus	0.2727	0.2232	0.2816	0.2225	0.3089	0.2731	0.3045	0.338	0.504
Bouboui virus	0.2889	0.2297	0.2698	0.2116	0.3351	0.3785	0.2452	0.2867	0.495
Bussuquara virus	0.2842	0.2174	0.283O	0.2155	0.3601	0.2576	0.3067	0.3066	0.499
Cell fusing agent virus	0.2443	0.2459	0.2744	0.2355	0.3115	0.3796	0.2407	0.2843	0.522
Chaoyang virus	0.2901	0.225	0.2704	0.2126	0.33	0.3704	0.2491	0.3071	0.495
Culex flavivirus	0.2349	0.2357	0.2973	0.2321	0.2501	0.2545	0.397	0.3328	0.529
Dengue virus 1	0.3191	0.2142	0.26	0.2087	0.3547	0.3652	0.234	0.3045	0.476
Dengue virus 2	0.3317	0.2111	0.2529	0.2043	0.4714	0.2334	0.2451	0.2871	0.457
Dengue virus 3	0.3219	0.2141	0.2586	0.2053	0.4462	0.2457	0.2693	0.2763	0.464
Dengue virus 4	0.3105	0.2201	0.2631	0.2063	0.4249	0.2473	0.2757	0.2825	0.47
Donggang virus	0.2707	0.245	0.2719	0.2124	0.3152	0.3769	0.2511	0.3051	0.501
Edge Hill virus	0.2847	0.2389	0.2842	0.1921	0.3288	0.3993	0.2482	0.2822	0.491
Entebbe bat virus	0.2783	0.2159	0.2806	0.2252	0.3473	0.383	0.2352	0.279	0.516
Gadgets Gully virus	0.2624	0.2166	0.313	0.2079	0.2986	0.3771	0.2743	0.2757	0.532
Hanko virus	0.2691	0.2657	0.2632	0.2019	0.3305	0.3314	0.3091	0.2726	0.465
Ilheus virus	0.2689	0.2082	0.2797	0.2432	0.3231	0.3587	0.2462	0.3097	0.535
Japanese encephalitis ORF	0.2776	0.2082	0.2836	0.2306	0.3342	0.2401	0.2989	0.3489	0.516
Jugra virus	0.2901	0.2282	0.2698	0.2119	0.3328	0.3843	0.2382	0.2915	0.493
Kadam virus	0.2547	0.22	0.3107	0.2156	0.3057	0.3668	0.286	0.2733	0.539
Kamiti River virus	0.2514	0.2487	0.2659	0.234	0.3214	0.3984	0.2325	0.2717	0.511
Karshi virus,	0.2431	0.2135	0.3211	0.2222	0.3026	0.3702	0.2723	0.2739	0.555
Kedougou virus	0.2431	0.2135	0.3211	0.2222	0.2587	0.2401	0.3928	0.3261	0.544
Kokobera virus	0.2813	0.2252	0.2892	0.21	0.3492	0.2586	0.3173	0.3049	0.496
Langat virus	0.2447	0.212	0.3231	0.2202	0.2897	0.3706	0.2812	0.2773	0.555
Louping ill virus	0.2447	0.2072	0.3213	0.2267	0.2695	0.2319	0.3766	0.3314	0.548
Meaban virus	0.2444	0.2136	0.3308	0.2112	0.3005	0.3715	0.2927	0.2632	0.556
Mercadeo virus	0.2506	0.2437	0.2758	0.2299	0.2708	0.2793	0.3449	0.3498	0.506
Modoc virus	0.2962	0.2516	0.2713	0.1809	0.3502	0.4155	0.2285	0.2741	0.467
Montana myotis leukoencephalitis virus	0.2981	0.2634	0.2602	0.1783	0.3849	0.3809	0.2558	0.2181	0.439
Mosquito flavivirus	0.2384	0.2395	0.2781	0.2435	0.3186	0.385	0.2311	0.2708	0.534
Murray Valley encephalitis virus	0.2888	0.23	0.2748	0.2114	0.3319	0.3763	0.2476	0.3041	0.498
New Mapoon virus	0.2635	0.2213	0.2706	0.2446	0.3134	0.365	0.2549	0.2954	0.53
Nounane virus	0.2856	0.217	0.2725	0.2245	0.367	0.2474	0.2875	0.3315	0.497
Ntaya virus	0.2856	0.217	0.2725	0.2245	0.3326	0.3788	0.2503	0.2914	0.493
Ochlerotatus caspius flavivirus	0.2688	0.2588	0.2665	0.2059	0.3354	0.4017	0.2326	0.2797	0.483
Omsk hemorrhagic fever virus	0.2566	0.2082	0.3127	0.2224	0.3058	0.3668	0.2723	0.2706	0.545
Palm Creek virus i	0.2636	0.2411	0.2878	0.2075	0.344	0.263	0.3588	0.2686	0.495
Paraiso Escondido virus	0.2896	0.2355	0.2899	0.1851	0.3286	0.3904	0.2579	0.2847	0.487
Parramatta River virus	0.2671	0.2595	0.2642	0.2093	0.3359	0.3938	0.2329	0.2816	0.485
Phnom Penh bat virus	0.2871	0.2663	0.2716	0.1748	0.3384	0.4136	0.2593	0.2664	0.461
Powassan virus	0.2516	0.2157	0.3128	0.2198	0.3052	0.369	0.2753	0.2685	0.545
Quang Binh virus	0.2388	0.2385	0.2776	0.2451	0.2615	0.2645	0.3321	0.3681	0.523
Rio Bravo virus	0.3025	0.2656	0.2502	0.1817	0.4023	0.3798	0.2283	0.2363	0.432
Saboya virus	0.285	0.2385	0.2706	0.2059	0.3285	0.3872	0.2449	0.2944	0.492
Saumarez Reef virus	0.2511	0.215	0.3173	0.2166	0.2973	0.374	0.2959	0.267	0.548
Sepik virus	0.2899	0.2386	0.2638	0.2077	0.3407	0.3931	0.237	0.2877	0.483
Spanish goat encephalitis virus	0.2442	0.2081	0.3233	0.2244	0.2982	0.3667	0.2776	0.273	0.559
Spondweni virus	0.2442	0.2081	0.3233	0.2244	0.3183	0.3631	0.2483	0.3024	0.532
St. Louis encephalitis virus	0.2918	0.2116	0.2801	0.2165	0.3399	0.3721	0.2471	0.293	0.507
Tamana bat virus	0.3312	0.2839	0.2156	0.1692	0.4208	0.4285	0.1901	0.2618	0.402
Tembusu virus	0.2866	0.2238	0.2894	0.2002	0.3304	0.3717	0.2567	0.2909	0.503
Tick-borne encephalitis virus	0.247	0.212	0.3207	0.2202	0.3013	0.3668	0.2743	0.2765	0.552
Uganda S virus	0.247	0.21	0.3207	0.2202	0.3802	0.3114	0.2594	0.2795	0.469
Usutu virus	0.2707	0.2192	0.2819	0.2281	0.3117	0.2699	0.3012	0.3368	0.511
Wesselsbron virus	0.2856	0.2395	0.2665	0.2085	0.3406	0.3906	0.2345	0.2926	0.487
West Nile virus lineage 1	0.2724	0.2155	0.2883	0.2238	0.3261	0.369	0.2552	0.2923	0.522
West Nile virus lineage 2	0.2727	0.217O	0.2827	0.2275	0.3141	0.2635	0.307	0.3417	0.51
Yaounde virus	0.2884	0.2068	0.2849	0.2199	0.3712	0.2188	0.3192	0.3227	0.505
Yellow fever virus	0.2704	0.2326	0.286	0.2109	0.3336	0.3969	0.2386	0.2839	0.509
Yokose virus	0.3003	0.2298	0.2597	0.2103	0.354	0.3887	0.2234	0.2839	0.483
Zika virus	0.2777	0.2146	0.2908	0.2169	0.3192	0.3704	0.2532	0.2952	0.52
average value	0.2732	0.2288	0.2849	0.21479	0.3310	0.3440	0.2705	0.2924	0.5052
SD	0.0236	0.0192	0.02532	0.0163	0.04052	0.0588	0.0410	0.02749	0.03233

**Table 3:** Optimal codons.

	Phe	Leu	Ile	Val	Ala	Thr	Pro	Ser	Tyr	TER	His	Gln	Asn	Lys	Asp	Glu	Cys	ARG	GLY
AEDES	1.27	1.64	1.26	1.39	1.20	1.23	1.30	1.04	1.45	0.58	1.19	1.32	1.18	1.42	1.10	1.26	1.29	1.92	1.13
Alkhurma virus	1.26	1.63	1.25	1.34	1.56	1.34	1.44	0.77	1.26	0.69	1.16	1.13	1.11	1.05	1.00	1.05	1.13	2.07	1.22
Apoi virus	1.11	1.49	1.70	1.48	1.35	1.43	1.67	1.16	1.28	0.51	1.15	1.39	1.08	1.19	1.16	1.20	1.33	1.66	1.46
Bagaza virus	1.13	1.89	1.41	1.37	1.52	1.49	1.26	0.81	1.21	0.56	1.16	1.13	1.13	1.13	1.24	1.40	1.37	2.44	1.71
Banzi virus	1.04	1.92	1.51	1.56	1.28	1.64	1.64	1.46	1.09	3.00	1.12	1.35	1.24	1.04	1.05	1.02	1.20	2.01	1.35
Bouboui virus	1.31	1.50	1.29	1.47	1.58	1.29	1.46	1.12	1.33	0.73	1.11	1.29	1.10	1.15	1.28	1.17	1.17	2.33	1.96
Bussuquara virus	1.12	1.69	1.17	1.70	1.29	1.46	1.49	1.54	1.01	3.00	1.08	1.03	1.06	1.12	1.19	1.18	1.06	1.34	1.38
Cell fusing agent virus	1.23	1.74	1.29	1.50	1.26	1.24	1.43	1.41	1.16	0.69	1.23	1.18	1.07	1.67	1.11	1.36	1.16	1.68	1.35
Chaoyang virus	1.24	1.58	1.27	1.36	1.43	1.19	1.48	0.90	1.19	0.60	1.14	1.24	1.12	1.27	1.08	1.30	1.08	1.34	1.83
Culex flavivirus	1.19	1.71	1.49	1.97	1.41	1.33	1.25	1.31	1.51	3.00	1.34	1.17	1.35	1.39	1.30	1.07	1.04	2.22	1.50
Dengue virus 1	1.25	1.65	1.19	1.66	1.95	1.35	1.57	1.00	1.46	0.46	1.04	1.26	1.09	1.28	1.11	1.32	1.09	3.35	2.29
Dengue virus 2	1.10	1.32	1.16	1.68	1.63	2.05	2.36	2.00	1.19	3.00	1.16	1.22	1.09	1.25	1.19	1.37	1.09	3.26	2.01
Dengue virus 3	1.24	1.34	1.14	1.73	1.35	1.83	1.85	2.07	1.00	3.00	1.00	1.05	1.22	1.24	1.12	1.26	1.08	3.08	2.06
Dengue virus 4	1.32	1.41	1.25	1.46	1.23	1.09	1.38	1.49	1.40	1.77	1.27	1.46	1.11	1.09	1.12	1.30	1.01	1.53	1.25
Donggang virus	1.30	1.73	1.36	1.58	1.75	1.24	1.48	1.80	1.03	1.87	1.24	1.34	1.28	1.01	1.11	1.04	1.31	2.06	1.13
Edge Hill virus	1.09	1.81	1.46	1.66	1.54	1.38	1.53	2.14	1.03	2.07	1.19	1.24	1.04	1.20	1.59	1.45	1.01	1.59	1.50
Entebbe bat virus	1.09	1.49	1.50	1.45	1.75	1.35	1.41	2.00	1.15	0.54	1.15	1.10	1.12	1.04	1.11	1.03	1.18	1.90	1.19
Gadgets Gully virus	1.09	2.15	1.23	1.47	1.47	1.11	1.84	1.75	1.02	3.00	1.21	1.04	1.04	1.06	1.02	1.22	1.25	1.80	1.93
Hanko virus	1.25	1.70	1.16	1.38	1.27	1.47	1.41	1.17	1.16	2.20	1.14	1.45	1.09	1.10	1.05	1.27	1.02	1.33	1.37
Ilheus virus	1.04	1.68	1.26	1.63	1.22	1.23	1.29	1.36	1.27	2.33	1.01	1.08	1.22	1.08	1.24	1.14	1.06	2.07	1.80
Japaneseencephalitisorf	1.07	1.67	1.34	1.65	1.80	1.15	1.70	1.16	1.21	2.08	1.16	1.42	1.07	1.26	1.25	1.18	1.17	2.03	1.29
Jugra virus	1.14	1.68	1.59	1.48	1.33	1.31	1.54	1.30	1.22	2.17	1.23	1.03	1.00	1.00	1.03	1.16	1.11	1.67	1.11
Kadam virus	1.24	1.78	1.44	1.33	1.37	1.23	1.29	1.37	1.36	2.00	1.28	1.27	1.01	1.42	1.19	1.36	1.31	1.41	1.65
Kamiti River virus	1.17	1.73	1.68	1.45	1.53	1.44	1.26	1.07	1.57	2.38	1.24	1.06	1.06	1.04	1.06	1.15	1.11	1.47	1.12
Karshi virus,	1.02	1.99	1.36	2.28	1.26	1.46	1.30	1.27	1.12	3.00	1.02	1.38	1.09	1.31	1.33	1.30	1.08	2.14	1.50
Kedougou virus	1.01	1.74	1.04	1.92	1.33	1.27	1.61	1.51	1.12	3.00	1.19	1.05	1.14	1.10	1.26	1.22	1.14	2.48	1.91
Kokobera virus	1.13	1.61	1.22	1.64	1.55	1.30	1.67	1.75	1.17	2.21	1.16	1.07	1.05	1.04	1.14	1.01	1.18	1.97	1.12
Langat virus	1.12	2.27	1.36	2.00	1.16	1.29	1.45	1.48	1.41	3.00	1.24	1.26	1.32	1.19	1.21	1.32	1.00	1.72	1.49
Louping ill virus	1.13	1.77	1.34	1.53	1.42	1.26	1.57	1.81	1.29	2.02	1.20	1.03	1.08	1.10	1.00	1.07	1.24	1.51	1.16
Meaban virus	1.12	1.46	1.50	1.39	1.16	1.21	1.21	1.26	1.24	3.00	1.27	1.07	1.43	1.06	1.06	1.09	1.17	1.10	1.96
Mercadeo virus	1.17	1.47	1.47	1.81	1.85	1.37	1.55	1.83	1.01	1.84	1.24	1.32	1.09	1.14	1.25	1.35	1.28	2.49	1.28
Modoc virus	1.36	1.77	1.37	1.55	1.69	1.52	1.94	1.83	1.09	3.00	1.32	1.09	1.21	1.14	1.24	1.40	1.32	2.98	1.73
Montana myotis leukoencephalitis virus	1.05	1.91	1.33	1.67	1.54	1.30	1.29	1.28	1.23	2.01	1.13	1.42	1.05	1.41	1.22	1.51	1.09	1.49	1.36
Mosquito flavivirus	1.29	1.83	1.45	1.40	1.65	1.37	1.35	2.17	1.05	2.13	1.08	1.11	1.10	1.19	1.10	1.30	1.07	1.89	1.40
Murray Valley encephalitis virus	1.02	1.54	1.33	1.19	1.66	1.26	1.87	1.88	1.65	2.00	1.19	1.24	1.01	1.25	1.03	1.15	1.13	1.64	1.50
New Mapoon virus	1.21	1.75	1.19	1.70	1.17	1.37	1.57	2.32	1.28	3.00	1.15	1.17	1.34	1.05	1.33	1.19	1.10	1.82	1.50
Nounane virus	1.47	1.64	1.39	1.60	1.57	1.18	1.32	2.13	1.27	2.02	1.11	1.13	1.09	1.32	1.17	1.25	1.10	1.95	1.53
Ntaya virus	1.26	1.71	1.30	1.56	1.33	1.30	1.44	1.53	1.28	2.04	1.19	1.56	1.07	1.07	1.27	1.36	1.24	1.44	1.48
Ochlerotatus caspius flavivirus	1.20	1.61	1.23	1.48	1.80	1.40	1.63	1.74	1.00	2.17	1.16	1.07	1.02	1.16	1.04	1.02	1.15	1.85	1.14
Omsk hemorrhagic fever virus	1.05	2.04	1.05	1.94	1.28	1.52	1.79	1.50	1.02	3.00	1.23	1.05	1.22	1.08	1.01	1.02	1.22	1.33	2.28
Palm Creek virus	1.26	1.66	1.47	1.76	1.22	1.34	1.42	1.92	1.49	1.98	1.36	1.14	1.11	1.23	1.21	1.18	1.10	1.78	1.31
Paraiso Escondido virus	1.32	1.85	1.29	1.76	1.08	1.12	1.35	1.48	1.33	1.99	1.21	1.57	1.12	1.41	1.15	1.53	1.14	1.40	1.26
Parramatta River virus	1.18	1.63	1.64	1.57	1.81	1.55	1.21	1.78	1.14	1.84	1.01	1.30	1.16	1.03	1.31	1.26	1.28	2.31	1.29
Phnom Penh bat virus	1.22	1.81	1.24	1.37	1.72	1.31	1.26	1.79	1.00	2.17	1.18	1.03	1.08	1.13	1.07	1.09	1.27	2.06	1.15
Powassan virus	1.19	1.58	1.72	1.42	1.41	1.35	1.09	1.17	1.41	3.00	1.36	1.06	1.32	1.18	1.22	1.04	1.04	1.52	1.66
Quang Binh virus	1.09	1.60	1.35	1.47	1.81	1.45	1.89	2.03	1.16	3.00	1.34	1.26	1.10	1.31	1.29	1.31	1.36	2.75	2.20
Rio Bravo virus	1.22	1.70	1.33	1.30	1.45	1.30	1.56	1.58	1.30	1.87	1.26	1.34	1.02	1.23	1.10	1.09	1.15	2.13	1.19
Saboya virus strain	1.16	1.77	1.19	1.51	1.49	1.45	1.46	1.57	1.23	2.03	1.27	1.03	1.03	1.13	1.11	1.10	1.27	1.75	1.27
Saumarez Reef virus	1.18	1.73	1.30	1.62	1.58	1.25	1.39	1.67	1.03	1.96	1.31	1.44	1.11	1.23	1.06	1.27	1.13	2.26	1.21
Sepik virus	1.38	1.70	1.08	1.42	1.71	1.47	1.38	1.59	1.10	2.25	1.05	1.06	1.02	1.15	1.04	1.01	1.17	1.17	1.12
Spanish goat encephalitis virus	1.12	1.53	1.20	1.39	1.68	1.28	1.45	1.82	1.23	2.38	1.14	1.16	1.19	1.35	1.13	1.19	1.11	1.41	1.23
Spondweni virus	1.25	1.93	1.57	1.42	1.51	1.42	1.49	2.25	1.08	2.25	1.15	1.26	1.06	1.26	1.18	1.35	1.14	1.79	1.36
St. Louis encephalitis virus	1.30	1.42	1.80	1.59	1.45	1.78	1.63	1.96	1.01	1.55	1.29	1.62	1.27	1.33	1.26	1.42	1.43	3.07	1.35
Tamana bat virus	1.36	1.74	1.52	1.33	1.55	1.43	1.29	1.87	1.13	1.79	1.33	1.10	1.01	1.32	1.02	1.13	1.22	1.92	1.18
Tembusu virus	1.19	1.74	1.55	1.38	1.66	1.38	1.38	1.68	1.11	2.34	1.10	1.01	1.01	1.09	1.01	1.03	1.17	2.01	1.10
Tick-borne encephalitis virus	1.11	1.51	1.16	1.47	1.36	1.06	2.25	2.07	1.14	1.14	1.19	1.26	1.07	1.17	1.05	1.15	1.12	2.44	1.70
Uganda S virus	1.03	1.74	1.21	1.43	1.25	1.40	1.59	1.38	1.17	1.50	1.26	1.15	1.22	1.11	1.21	1.09	1.00	2.25	1.73
Usutu virus	1.11	1.60	1.45	1.55	1.59	1.32	1.32	1.68	1.16	2.02	1.22	1.32	1.32	1.32	1.13	1.30	1.04	2.16	1.30
Wesselsbron virus	1.23	1.55	1.57	1.47	1.70	1.32	1.46	1.71	1.02	2.35	1.00	1.14	1.10	1.24	1.28	1.27	1.12	1.69	1.27
West Nile virus lineage 1	1.08	1.81	1.42	1.67	1.37	1.26	1.62	1.52	1.26	3.00	1.25	1.10	1.10	1.08	1.12	1.05	1.16	2.01	2.03
West Nile virus lineage 2	1.13	1.77	1.50	2.03	2.03	1.49	1.49	1.70	1.22	3.00	1.18	1.09	1.29	1.04	1.23	1.26	1.16	2.18	2.30
Yaounde virus	1.13	1.56	1.27	1.44	1.64	1.29	1.62	1.82	1.20	2.28	1.28	1.16	1.18	1.20	1.14	1.27	1.16	1.87	1.37
Yellow fever virus	1.31	1.57	1.44	1.75	1.40	1.25	1.50	1.79	1.28	2.03	1.15	1.33	1.00	1.31	1.10	1.41	1.17	2.01	1.37
Yokose virus	1.02	1.62	1.24	1.56	1.61	1.46	1.48	1.84	1.23	2.02	1.21	1.21	1.03	1.14	1.10	1.13	1.09	2.00	1.38
Zika virus	1.08	1.42	1.22	1.29	1.62	1.07	1.32	1.13	1.00	3.00	1.05	1.23	1.16	1.23	1.33	1.07	1.04	1.19	1.57

Ration of A/U-ended codons(%)	84.62	61.45	76.92	63.07	84.62	81.54	98.46	76.92	69.23	84.62	87.69	73.85	46.15	78.46	64.61	83.82	78.46	83.07	93.85

**Table 4:** Correlation analysis.

Variables	A	U	G	C	GC	Gravy	Aromo	ENC
U_3s_	0.05	0.374*∗∗*	-0.09	-0.350*∗∗*	-0.078	-0.793*∗∗*	-0.157	0.173
C_3s_	-0.18	-0.294*∗*	-0.006	0.565*∗∗*	0.23	0.384*∗∗*	0.13	0.256*∗*
A_3s_	0.822*∗∗*	0.158	-0.575*∗∗*	-0.531*∗∗*	-0.759*∗∗*	0.133	0.23	-0.752*∗∗*
G_3s_	-0.473*∗∗*	-0.279*∗*	0.431*∗∗*	0.345*∗∗*	0.404*∗∗*	0.628*∗∗*	0.118	0.14
GC_3s_	-0.471*∗∗*	-0.380*∗∗*	0.358*∗∗*	0.563*∗∗*	0.462*∗∗*	0.580*∗∗*	0.059	0.264*∗*
ENC	-0.757*∗∗*	-0.15	0.442*∗∗*	0.640*∗∗*	0.710*∗∗*	-0.279*∗*	-0.333*∗∗*	

Note: *∗∗*Means p < 0.01.

*∗*Means 0.01 < p < 0.05.

N Means no correlation.

## Data Availability

The data used to support the findings of this study are included within the article.
